# Hospital Meetings

**Published:** 1901-04-13

**Authors:** 


### The Institutional Workshop.

### HOSPITAL MEETINGS.

### ROYAL EYE HOSPITAL.

The forty-fourth annual meeting of the Royal Eye Hospital, St. George's Circus, Southwark, was held on March 27th, Professor McHardy presiding. In moving the adoption of the report and accounts, he said that Mr. W. Moresby Chinnery, their President, had intended to take the chair, but had been prevented at the last moment from coming. In looking through the report, the Governors would note with pleasure that the King, who as Prince of Wales, was for many years one of their patrons, and who laid the foundation-stone of the building, had consented to continue his patronage. During the year they had treated 632 in-patients, and 17,529 out-patients (whose attendances numbered 54,507), totalling together 18,161, as compared with 18,499 last year. This slight falling off in numbers was, he thought, mainly due to a little less want of consideration on the part of the School Board this year than last when, without warning, and during the holiday season, they sent the school-children, at the rate of 800 per week, to have their sight tested, obliging the hospital to employ three additional assistant surgeons. This, he was glad to say, had been a good deal mitigated during the past year. Their total income for the year, inclusive of £300 from legacies and £800 from the Banquet and Jubilee committees, was £3,321, and their total expenditure, including £172 for repairs, was £3,341. There was thus a difference of only £20 between receipts and disbursements, and it was the settled practice and policy of the Council to keep the two as nearly level as possible, feeling that, on the one hand, they ought not to hoard funds given them to combat blindness; and, on the other hand, that they ought not to run into debt any more in their corporate capacity than they would as individuals. The estimated cost of each in-patient averaged £2 2s., the average stay being 14·8 days. The average expenditure on out-patients worked out at less than 2s. 3d. The cost per head showed an increase on last year owing to the higher prices of gas, fuel, and other necessaries. Their results, he thought, compared very favourably with what was done anywhere else; and this he considered was due not only to the skill and care at present exercised, but also to the skill and care with which the building had been designed for its special purposes, and to the provision of proper appliances and conveniences for carrying on the work. They were thankful to the Prince of Wales's Fund for their donation of £150, but he could not help noticing that another similar institution, treating less than double their number of patients, got £1,200. It seemed to him that either the Royal Eye Hospital should have received £600, or else that the hospital he referred to should only have had £300. One item in the receipts they would notice was payments by in-patients, £365. That revenue was derived from the four private rooms reserved for a class of patients able to pay for their maintenance, and where they could have better nursing and care than in any hotel or private house, and where also there were no corners, and no blinds and curtains to hold that dust which was such a cause of terror to the ophthalmic surgeon. For these rooms they received three guineas a week or more, and as they only cost one guinea there was a substantial profit. But these private rooms were also of great importance in bringing in a new class of people and so getting them new governors, and as a matter of fact many of their new governors and subscribers were connected with their patients. It was a matter of regret that no memorial beds had been added during the past year; but it was hoped that some of those bereaved by the war or otherwise would take this wise and large-hearted means of honouring the memory of departed friends. The next annual festival dinner was to be held on July 8th, when Sir John Blundell Maple had promised to preside. The position of the hospital was their great reason for holding annual festivals, for though it was the best possible position for the work, it was far removed from the ken of the well-to-do, whom by that means they were able to approach. Their building stood at the geographical centre of London, and where six main roads met, some thousands of 'buses and tramcars passing daily. And though next to the wealthy parish of St. Saviour's, Southwark, their own parish of St. George's was the poorest in all London. It was believed that six out of ten of its inhabitants went hungry from Christmas to Christmas, and it was so terribly overcrowded that it was almost impossible to get even a lodging in it. The Chairman referred to the coming removal of the School for the Indigent Blind from the opposite side of the Circus as meaning a possible loss of income to the hospital, since while it stood there they had been able to say to people, Give 1s. to the Eye Hospital, and so save the spending of £1 by the Blind School. He mentioned that Sir Henry Irving had promised to lend the Lyceum for a matinée in aid of the hospital, and that Miss Grainger Kerr was again getting up a concert for them. The honorary staff had been augmented by the appointment of Mr. Brooksbank James, F.R.C.S., and Mr. A. Ormond, F.R.C.S., as assistant surgeons, and the out-patient department would in future be open both in the forenoon and afternoon, thus stretching the capacity of the hospital without further expense on premises. He concluded by referring with regret to the death on the 17th of the month of Mr. G. W. Barnard, who for upwards of a quarter of a century had been their honorary solicitor.

The report was adopted, and motions for the election of Lord Llangallock as a vice-president, for the re-election of the ten members of the council, and of thanks to the staff having been agreed to, the proceedings terminated.

### POPLAR HOSPITAL FOR ACCIDENTS.

The annual general meeting of Governors of Poplar Hospital for Accidents was held on Tuesday, 26th inst., the Hon. Sydney Holland presiding.

The Chairman dealt first with the statement of accounts. The ordinary income amounted to £8,810, while the ordinary expenditure was £6,578; that was to say an average of £20 per day. This amount had by some means to be collected. The hospital was supported in a very loyal way: the record of £750 from the working men was one almost unprecedented. The total amount could not appear in the balance-sheet, as the result of Mr. Relf's music-hall entertainment was received too late for inclusion. The working men's contributions had increased by £200 since 1889. He especially wished to congratulate the Canning Town men on the revival of their interest in the hospital. Patients' payments amounted to £131; these payments were for dressings, and were very small. He had never known a patient who claimed that he was unable to pay the twopence for bandages. No payments were taken from children. Probationers' fees came to £100. The probationers were not asked to pay unless they could afford it, or came for a very short time. Two items of which he was sure the Governors would approve, appeared on the expenditure side; viz., payments to the family of a porter absent in South Africa, and a pension to an old lady who had scrubbed in the hospital for twenty years. Turning to the report, the chairman said the cost of building the new medical wing, including furnishing, worked out at £20,000.

The Drapers' Company had given £14,200, leaving a balance to be raised of £6,083. Towards this deficiency the Prince of Wales's Fund had given £500, and the Corporation of the City of London £210. This left £5,373 to be found. The policy of the hospital was "never to be in debt"; and as it would be a risk to have no reserve fund, they did not wish to touch that fund unless to prevent a debt. The number of in-patients was 954, as compared with 877 in 1899. The out-patients numbered 23,742, the largest number on record. Of these comparisons, however, he did not think much, as circumstances controlled the numbers in a hospital for accidents. They were grateful to Mr. W. Crooks, L.C.C., for persuading the Council to lay out the waste land opposite the hospital, and also to the East India Company for giving up a piece of road for the same purpose. Mr. Whyte, who had held an important position at the London Hospital, had been elected senior resident surgeon; he was both careful and sympathetic in his treatment, and there was every reason for confidence that he would give satisfaction to the patients. The report and balance-sheet, as proposed by Mr. H. J. Lefeaux and seconded by Mr. Piper, having been adopted, the officers for the year were elected, and the meeting terminated with the usual votes of thanks.

### NATIONAL HOSPITAL FOR DISEASES OF THE HEART.

The annual general meeting of the National Hospital for Diseases of the Heart was held on Wednesday, 27th inst., the Right Hon. the Earl of Verulam presiding. The report for 1900 showed that the income of the general fund from all sources was £1,785, and the expenditure £2,151, as against £1,995 and £2,448 respectively in 1899. The committee regretted that, owing to the urgent appeals made for the numerous war funds, a considerable number of donations, upon which they had been accustomed to rely, had greatly fallen off. They had, however, received from the Hospital Sunday and Saturday Funds £76 13s. 4d. and £54 12s. respectively; from the Leathersellers' Company, £10 10s.; Messrs. Marshall and Snelgrove, £10 10s.; and £50 from other donors. There had been 13,189 attendances of out-patients, as against 13,020 in the previous year. On account of the alterations it had been necessary to close the wards for in-patients for a considerable portion of the year, but there had been no interruption in the treatment of out-patients. New bath-rooms and lavatories, with the latest and best sanitary appliances, had been erected, the heating and ventilating of the wards had been greatly improved, and several other extensive alterations that were urgently required, and which would conduce greatly to the comfort and benefit of the patients, had been carried out. The best thanks of the committee were tendered to the Hon. Medical Staff for the zeal and public spirit shown in attending the out-patients during the alterations, which rendered their duties both inconvenient and difficult. Lord Verulam, in moving the adoption of the report and accounts, said they might congratulate themselves most particularly upon the fact that the funds had been sufficient to meet the cost of the alterations. He had taken the opportunity of going over the hospital, and had been much impressed with the good quality of the work; the solidity, and what he might call the strategic value of it seemed to be calculated to save as far as possible the labours of the nurses and medical staff. He thought they might anticipate a successful year for the hospital. W. Miles Booty, Esq., seconded; he also spoke of the devotion of the medical staff to their work, and the ardour with which they continued it in the midst of the alterations. The report and accounts were unanimously adopted. Dr. Gibbes replied; he thought that the harmony existing between the medical staff and the committee could not be better shown than in the way in which the change of name of the hospital had been carried out. There would soon be another revolution in the revision of the rules; he hoped it would be carried out in the same peaceable way. The usual votes of thanks closed the meeting.

### LONDON HOMŒOPATHIC HOSPITAL.

Earl Cawdor presided at the fifty-first annual general meeting of the London Homœopathic Hospital on the 28th ult., which was a well-attended gathering, many ladies being present despite the inclement weather.

The Chairman, in moving the adoption of the report, said they were glad to have been some use in the war; the Board had felt they were only carrying out the wishes of the Governors in giving permission to five of their best nurses to join the Army Nursing Corps, though necessarily it had been at the cost of some inconvenience. He referred to the award of £200 from the Prince of Wales's Fund as a satisfactory feature in their accounts, since as they all knew such awards were made to them solely on the merits of the work done. Naturally they were glad that their income had again increased on that of the previous year, while their expenditure had remained stationary. He had ventured to predict last year, with the concurrence of their able secretary, that their expenses had reached their limit, and had become about normal. This year they did show a decrease in the items they were able to control, the increase being in the items affected directly by the increased work done. He thought their annual subscriptions were not proportionate to their expenses, the comparison being in round figures £1,500 and £10,000, and that it would be wise to get that proportion increased by persuading all their friends to increase—perhaps to double—their subscriptions so as to establish equilibrium between their income and outlay, and so avoid further trenching upon their invested capital.

Mr. Stillwell, the chairman of the Board of Management, seconded. He thought, considering the admittedly high state of efficiency maintained, that they might congratulate themselves on the item for management in their expenditure account being only £677. It was generally considered that from 10 per cent. to 15 per cent. was a fair expenditure in a hospital which had 100 beds, as they had, and he believed that was about the average in institutions of about the same size as theirs. He therefore thought it was peculiarly to their credit to find that they were being run with an expense of only 6·9—under 7 per cent.—on management. So far as he knew there was only one hospital, and that a very large one, namely Guy's, which could say the same. He thought it something they might be proud of that only that huge London hospital, with its 600 beds, was able to bring down its management expenses to the same level as themselves. With reference to the question of annual subscriptions he was happy to be able to announce that Lord Cawdor had told him of his intention to raise his to £25; he (the speaker) would follow by doubling his own, and by trying what could be done with all his friends. He concluded by a warm expression of appreciation of the services of their secretary, Mr. Cross, and his assistant.

The report was adopted, and it was then proposed that Law 50, which provided that—

"All applications for Medical Appointment on the Honorary Acting Medical Staff, excepting for those of Consulting Physicians, Consulting Surgeons, or Salaried Medical Officers, shall be submitted to the Medical Council for their report to the Board. The appointment to the Medical Staff shall take place at a meeting of the Board of Management, to which meeting the members of the Medical Council and the Medical Staff shall have been convened to advise the Board. But if, in the opinion of the Board, there is no

Candidate of sufficient merit for the vacant post, no election shall take place and the vacancy shall be again advertised" should be altered by the omission of the words "to which meeting the members of the Medical Council and the Medical Staff shall have been convened to advise the Board."

Mr. Stilman said the Medical Council always advised the Board as to appointments and that advice was always accepted—unless indeed there were obvious reasons to the contrary—so that it was not necessary for them to give the same opinion over again. Sir Henry Tyler, in seconding, observed that the provision now to be omitted was clearly unnecessary, since as a matter of fact the medical men, though invited, never came on those occasions.

Dr. Galley Blackley, one of the physicians, moved a vote of thanks to the Board, the Officers, the House Committee, and the Visitors. He spoke in the warmest terms of the cordial relations existing between the management and the staff, and complimented the Board on the early adoption of the idea of having two members of the staff elected each year to the Board. From the very beginning the plan had proved an unqualified success. It was a great pity that years ago it had not been adopted elsewhere; it would have saved the unhappy controversy that, in their immediate neighbourhood, had been going on for so many months. He thought they might fairly claim that was one of the ways in which they had "shown the way"—another being that they were the first hospital to appoint ladies to residential positions.

Dr. Byres Moir, another of the physicians, seconded the resolution in terms equally cordial, and mentioned that the fame of the London Homœopathic had gone far and wide, he having heard just recently of a lady who, having been commissioned to inspect and report on various hospitals with the view to efficiency in the building of a new one had, after going over a very well-appointed one in America which pleased her very much, was told there were believed to be only two to beat it—a new one in Paris, and the London Homœopathic.

The motion was carried by acclamation.

Sir Henry Tyler, who moved the re-election of the medical staff, and other speakers, also dwelt on the extremely harmonious relations, the hearty co-operation, and the brotherly union existing between the Board and the medical staff.

### ROYAL NATIONAL HOSPITAL FOR CONSUMPTION.

The thirty-second annual meeting of the Royal National Hospital for Consumption and Diseases of the Chest, Ventnor, Isle of Wight, was held on March 28th at the Offices, 34 Craven Street, Charing Cross, W.C. It was expected that Lord Alverstone would preside, but his lordship was detained in the Upper House, and the chair was taken by Mr. P. B. Burgoyne, the deputy-chairman and treasurer.

The report of the Board of Management stated that the number of in-patients under treatment during the year was 899, and there were 247 candidates on the register waiting for admission at the end of the year. The average number of beds daily occupied was 139, compared with 129 in the previous year. The mean duration of each patient's residence in the hospital was 67 days, against 64 days in 1899. In order to provide better quarters for the resident medical staff certain rearrangements have been made; but the number of beds has only been reduced by one—from 153 to 152. The out-patient department has been practically abolished by the substitution for the old regulation of one whereby accepted patients who are waiting for admission into the hospital may attend as out-patients until their turns arrive, but are not visited at their own homes. Applicants are now accepted or rejected medically about one week before their turns for admission are expected to arrive. It is hoped that this will better limit the patients to those in the early or incipient stages of disease. The total expenditure was £16,441, made up of the following:—Ordinary expenses, £14,648; extraordinary expenses (for deepening the well, balance of the electric light installation, and for additional cooking apparatus), £892; and repayment of overdraft at bank from previous year, £900. There was a large increase in the cost of maintenance, the principal advances being:—Provisions, £663; lighting, in consequence of the adoption of electric light, £150; coals, £476; rates and taxes, in consequence of increased assessment from £585 to £1031, £46; and repairs £190. Including legacies, amounting to £478, the receipts were £13,248, which left a deficiency of £3,193. This had to be provided by the sale of the invested reserve fund to the extent of £3,000. The Hamilton Trust Funds were voted among 33 poor patients. After a service of 16 years as an examining physician in London, Dr. J. C. Thorowgood, F.R.C.P., resigned on his removal to the country, and accepted the appointment of consulting physician. Dr. Williamson resigned the post of a physician to the hospital in Ventnor, having been connected with the institution since 1873. Dr. E. Symes-Thompson, F.R.C.P., had accepted the post of physician in London. The two vacancies as physicians in Ventnor, one caused by the death of Dr. Coghill, had not yet been filled up. Dr. C. Holman had resigned his seat as a member of the Board of Management. The local management had been placed under a visiting committee in the place of the former house committee. Owing to the death of Major Payne, a vacancy arose in the office of general superintendent at Ventnor, and the Board had secured the services of a suitable successor.

The Chairman, in moving the adoption of the report, referred to the death of the Queen and to the interest which her Majesty took in the hospital. He said he was glad to be able to inform them that his Majesty King Edward VII. had graciously continued the late Queen's generous subscription. A petition to the King was being prepared and would, in due course, be presented, praying that his Majesty would continue the Royal patronage which had been of so much advantage to the hospital. Every bed was fully occupied during the year, and the number of patients treated had been enlarged to 899. It was sad that there were an increasing number of applicants for admission, and during the past year there had been about 240 usually waiting on the books. The income last year had unfortunately been insufficient for the maintenance, but although they were £3,192 short, he would not have them suppose that the hospital had lacked support. On the contrary, the public had been most generous, and thanks in a great measure to one handsome donation of £1,000, the receipts had been well maintained. The legacies had been but £478. There had been unusual extra expenses, due in a great measure to the enlargement of the hospital, to the increase in cost of coals, and to the new regimen which is now recommended for the patients, by which they are urged to take as much of the excellent food provided as they can eat. The cost of provisions had therefore been very largely increased. There had been other special expenses, notably electric light, for it was of the first importance that they should have pure air in the hospital.

The motion was duly seconded and carried.

Mr. P. B. Burgoyne was re-elected treasurer, and the retiring members of the Board of Management were re-appointed, Mr. Grosvenor Woods, K.C., of Ventnor, being added to the Board. Mr. William B. Peat, was re-elected auditor at a fee of £10 10s., which, the Chairman mentioned, Mr. Peat returned to the hospital. A vote of thanks to the Chairman concluded the proceedings.

DENTAL HOSPITAL OF LONDON.

Mr. Stuart Samuel, M.P., took the chair at the annual general meeting of the Dental Hospital of London on March 28th, in the handsome new building adjoining the old premises in Leicester Square, which is now practically ready for occupation. The report of the Committee of Management reviewed the circumstances leading up to the new departure: £42,000 was paid for the site, and very nearly £50,000 on the erection. The old building had been sold for £18,500. Shops form part of the ground floor of the new premises; two of these were let and would be occupied immediately. The site and buildings had been mortgaged for £55,000 at 3½ per cent., and the Charity Commissioners required that the debt shall be extinguished within thirty years. They also forbade the extinction of the license of the public house which, being originally on the site, now formed part of the new building; but it was proposed to raise a special fund for the purchase of the license with a view to its extinction, since it was felt that the presence of a public house forming part of a building devoted to the purposes of a public hospital could not but be open to very serious objection. The ordinary income for the past year amounted to £3,259, and the ordinary expenditure to £2,027, the difference, £1,207, being carried to the building account, the donations to which amounted to £2,632. Dr. Walker, the treasurer, having resigned owing to failing health, Mr. F. A. Bevan, one of the trustees, had accepted the post, which was formerly held for many years by his father, the late Mr. R. C. L. Bevan. The committee mentioned with regret the death of the Bishop of London, a vice-president; of Mr. Thos. Underwood, who served the hospital for many years; and of Mr. Storer Bennett, who was formerly on the staff, and at the time of his decease a member of the managing committee.

The report was adopted and resolutions agreed to reappointing the committee, appointing Mr. Bevan treasurer, and making verbal alterations in the laws relating to the signing of cheques.

The Chairman referred to the difference in the conditions and the work, now that they were able to meet in that magnificent new building, and those obtaining in 1874, when the number of operations performed was only 19,000 as compared with 69,000 last year; after which the meeting (which was held in the spacious theatre, capable of seating 300 students) separated to inspect the various parts of the new building.

THE BRITISH LYING-IN HOSPITAL.

The annual general meeting of Governors of the British Lying-in Hospital was held on Thursday, 28th ult., Mr. C. E. Farmer, in the chair. The Chairman referred in sympathetic terms to the death of the matron, Miss Mabel Cook; during the three years she had been matron the number of in-patients per year had risen from 187 to 354, while the out-patients had increased from 390 to 529. Through her exertions the hospital had been made much more attractive to poor people; she had met her death while at work, from blood-poisoning; there was always a risk of this, though the antiseptic treatment reduced it. He thought there was a great deal of heroism about the life of a nurse. Her place was being temporarily filled by Miss Knott, the assistant matron. He drew attention to the reconstruction of the drains, which had been carried out in accordance with the plans and under the personal superintendence of the London Sanitary Protection Association; the large sum of £417 4s. 2d. had been met by the income of the year; and 1900 ended for the hospital absolutely free of debt. The Ladies' Committee had established a Samaritan Fund, which was used for providing poor patients, on leaving the hospital, with boots, shawls, cab-fares, &c.; many were very badly off for comforts. The scheme for utilising a house in Betterton Street, owned by and adjacent to the hospital, for the purpose of a Nurses' Home, was still under consideration; if carried out it would have the effect of adding another ward, and so preventing overcrowding. The outlay would be something like £5,000. When sufficiently advanced, the scheme would be brought before the Governors and subscribers. During the year 26 midwifery pupils and 108 nurses had been trained; if the proposed alterations were carried out a yet larger number of nurses would be wanted; there ought to be a nurse for every bed. There was an item in the list of annual subscriptions to which he wished to call attention, viz. subscriptions in arrear; all had now been paid except about £5, but the Committee had been considering whether a rather earlier application for subscriptions would ensure the total omission of this item from the accounts.

Mr. A. H. P. Strickland, Treasurer, seconded, and the report and accounts were adopted.

